# No effect of short‐term exposure to ocean acidification on individual‐level variation in behaviour and susceptibility to predation in a Great Barrier Reef damselfish

**DOI:** 10.1111/1365-2656.70346

**Published:** 2026-10-08

**Authors:** Dominique G. Roche, Josefin Sundin, Ben Speers‐Roesch, Shinichi Nakagawa, Timothy D. Clark, Sandra A. Binning

**Affiliations:** ^1^ Fish Ecology and Conservation Physiology Laboratory, Department of Biology Carleton University Ottawa Ontario Canada; ^2^ Institut de Biologie, Éco‐Éthologie Université de Neuchâtel Neuchâtel Switzerland; ^3^ Department of Neuroscience Uppsala University Uppsala Sweden; ^4^ Department of Aquatic Resources Swedish University of Agricultural Sciences Drottningholm Sweden; ^5^ Department of Biological Sciences University of New Brunswick Saint John New Brunswick Canada; ^6^ School of Biological, Earth and Environmental Sciences University of New South Wales Sydney New South Wales Australia; ^7^ Department of Biological Sciences University of Alberta Edmonton Alberta Canada; ^8^ School of Life and Environmental Sciences Deakin University Geelong Victoria Australia; ^9^ Département de Sciences Biologiques Université de Montréal Montréal Québec Canada

**Keywords:** behavioural traits, coral reef fish, novel object test, open‐field test, personality, predator–prey interaction, repeatability, survival analysis

## Abstract

1. Ocean acidification, caused by rising carbon dioxide (CO_2_) in the atmosphere, has been reported to negatively impact a wide variety of behaviours in fishes, including activity, exploration, and predator avoidance.
2. These effects have been documented at the population level, but many animal species naturally show large and repeatable individual‐level differences in behaviour. How environmental stressors, such as ocean acidification, affect behavioural variation at the individual level remains largely unknown but is vital in understanding adaptation given natural selection operates on variation at the individual rather than population level.
3. Using a statistical approach allowing variation in means and variation in variance to be modelled within a single framework, we quantified individual‐level differences across five behaviours in the coral reef Ambon damselfish *Pomacentrus amboinensis* (emergence time, activity level, time spent sheltering, thigmotaxis and novel object inspection). We measured behaviour in a novel environment assay, twice before (CO_2_ ~450 μatm) and twice following 4–6 days of exposure to predicted end‐of‐century ocean acidification conditions (~1100 μatm).
4. Following behavioural assays, we tested individual survival in a live predation experiment. We used predatory rock cod, *Cephalopholis microprion*, acclimated to the same CO_2_ treatments as Ambon damsels and examined predictors of survival probability.
5. All behaviours in damselfish were moderately and significantly repeatable, with no marked differences in repeatability estimates between the ambient CO_2_ and elevated CO_2_ treatment groups. Exposure to end‐of‐century ocean acidification conditions had no effect on any of the five behaviours measured, both in terms of group means and residual (within‐individual) variance.
6. The probability of survival in the predation trials was similar for damselfish in the ambient and elevated CO_2_ treatment groups. Smaller damselfish as well as those that spent a greater amount of time inspecting a novel object (i.e. bolder individuals) had a lower probability of survival regardless of their CO_2_ treatment.
7. Our results challenge assumptions about the impacts of ocean acidification on coral reef fish behaviour and susceptibility to predation, both at the population and individual level. They also provide support for a trade‐off between boldness and predation risk in fish.

## Graphical abstract

This study shows that projected ocean acidification does not alter behaviour or predation risk in coral reef fish, challenging earlier assumptions. By quantifying consistent individual‐level behavioural differences and relating individual traits to survival during a live‐predation trial, it also provides evidence for a boldness–predation trade‐off in a small‐bodied demersal fish.

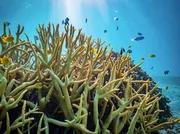

## Résumé

1. L'acidification des océans, causée par l'augmentation du dioxyde de carbone (CO_2_) atmosphérique, a été associée à des effets négatifs sur le comportement des poissons, notamment l'activité, l'exploration et l'évitement des prédateurs.
2. Ces effets ont principalement été étudiés à l'échelle des populations, alors que de nombreuses espèces présentent d'importantes différences interindividuelles répétables dans leur comportement. Comment les facteurs de stress environnementaux influencent cette variation individuelle demeure largement inconnue, bien qu'elle soit essentielle à notre compréhension de l'adaptation.
3. À l'aide d'une approche statistique permettant de modéliser simultanément les changements de moyenne et de variance, nous avons quantifié les différences interindividuelles dans cinq comportements chez le poisson‐demoiselle (*Pomacentrus amboinensis*): le temps d'émergence, le niveau d'activité, le temps passé à l'abri, la thigmotaxie et l'inspection d'un nouvel objet. Le comportement a été mesuré deux fois avant (CO_2_ ~ 450 μatm) et deux fois après une exposition à court terme aux conditions d'acidification prévues pour la fin du siècle (~1100 μatm).
4. Suite aux tests comportementaux, nous avons évalué la survie individuelle lors d'expériences de prédation avec le mérou *Cephalopholis microprion*, acclimaté aux mêmes conditions de CO_2_ que les poissons‐demoiselles, afin d'identifier les facteurs prédictifs de la survie.
5. Les cinq comportements présentaient une répétabilité modérée et significative, sans différence marquée entre les traitements de CO_2_ ambiant et élevé. L'exposition à court terme aux conditions d'acidification prévues pour la fin du siècle n'a eu aucun effet sur les comportements étudiés, tant sur les moyennes des groupes que sur la variance résiduelle intra‐individuelle.
6. La probabilité de survie lors des expériences de prédation était similaire entre les deux traitements de CO_2_. Les individus de plus petite taille et ceux consacrant davantage de temps à l'inspection d'un nouvel objet (c'est‐à‐dire plus téméraire), présentaient toutefois une probabilité de survie plus faible, indépendamment du traitement de CO_2_.
7. Nos résultats remettent en question certaines hypothèses concernant les effets de l'acidification des océans sur le comportement et la vulnérabilité à la prédation des poissons de récifs coralliens, tant à l'échelle populationnelle qu'individuelle. Ils appuient également l'existence d'un compromis entre les comportements téméraires et le risque de prédation chez les poissons.

## 1 INTRODUCTION

The partial pressure of carbon dioxide (CO_2_) in the oceans has increased by ~45% over the past century, driving a process known as ocean acidification (OA) (Hönisch et al., 2012; Lüthi et al., 2008). This environmental change shows no signs of stopping: forecasts predict that oceanic CO_2_ levels at the beginning of the next century will reach those not seen in the last 30 million years (Lüthi et al., 2008). Among the range of detrimental impacts reported for animals under ocean acidification, altered species interactions—including between predators and their prey—have received considerable attention (Draper & Weissburg, 2019). For example, lab‐based experiments have suggested that end‐of‐century levels of CO_2_ cause coral reef fishes to become highly attracted to the chemical cues of their predators (Munday et al., 2010), implying increased predation risk under these scenarios (but see Clark et al., 2020, which questions the repeatability of this finding). Indeed, damselfish exposed to end‐of‐century CO_2_ and then returned to their natural coral reefs were reported to disappear faster than control individuals (Munday et al., 2010, 2012). The exact fate of these fish was not recorded, but suggestions for their disappearance included predator attraction, increased activity/boldness, and poorer homing/sheltering ability, all leading to increased predation (Briffa et al., 2012; Devine et al., 2012; Devine & Munday, 2013; Munday et al., 2010). However, a limited number of studies have directly tested the hypothesis that ocean acidification leads to decreased prey survival during a predator interaction. Interestingly, the few laboratory studies on predator–prey interactions between coral reef fishes following short‐term (4–7 days) exposure to end‐of‐century levels of CO_2_ suggest that the performance of both predators and prey may be altered (e.g. Allan et al., 2013, 2017). Conversely, and contrary to early findings (e.g. Munday et al., 2010), there is increasing evidence that ocean acidification may have only negligible effects on coral reef fish behaviour (Clark et al., 2020; Clements et al., 2022; Sundin, 2023). Moreover, immediately prior to the publication of the current study, one of the foundational papers reporting behavioural effects of ocean acidification on fishes (Dixson, Munday and Jones, *Ecology Letters* 2010) was retracted (Retraction, 2026). The uncertainty in the field makes it difficult to predict the impact of future ocean acidification on predator–prey relationships on coral reefs.

Many experiments exploring fish behaviour following exposure to ocean acidification report extremely low inter‐individual variability in behavioural responses (e.g. Munday et al., 2010; but see Clements et al., 2022). This low variability is particularly alarming given that the resilience of a species to environmental perturbations depends on variation in individual responses (Tuomainen & Candolin, 2011). Phenotypic variation is an inherent biological trait that forms the basis for natural selection and enables an adaptive response to stressors across generations.

Individual behaviours that are repeatable across time and contexts are often referred to as animal ‘personality’ (Carter et al., 2013; Roche et al., 2016; Wolf & Weissing, 2012), and interest in understanding the implications of these individual‐level differences on trait evolution has grown over the last 20 years. Behavioural differences influence both the ways in which individuals interact with their environment as well as the outcome of biotic interactions, such as predation, competition, parasitism, cooperation and mate‐choice (Dubois & Binning, 2022; Gradito et al., 2024; Roche et al., 2016; Wolf & Weissing, 2012). For example, individuals that are consistently ‘bold’ may expend more energy on activity, increasing their susceptibility to predation but also providing greater foraging opportunities compared with ‘shy’ individuals that remain in hiding for longer durations. Thus, in order to understand how a species might respond to environmental stressors, including ocean acidification, variability in individual responses should be explicitly considered rather than overlooked.

Focusing on individual‐level variation as opposed to population mean responses to environmental perturbations has major conservation implications by allowing us to understand the traits responsible for conferring resilience to stressful conditions (Browman, 2016; Killen et al., 2016). As such, behavioural ecologists are increasingly adopting a reaction norm approach to the study of behavioural variation in order to examine how behaviour and phenotypic plasticity are correlated and/or under selection in a given environment (Dingemanse et al., 2010; Dingemanse & Dochtermann, 2013; Roche et al., 2016; Figure 1). A reaction norm is the range of phenotypes (behaviours, morphologies or physiological states) that an individual can express across time or a range of environmental conditions. Hypothetically, a population‐level increase in a behaviour (e.g. activity level) due to elevated CO_2_ exposure can occur from all individuals increasing their activity score (Figure 1a1), or only some individuals exhibiting a marked increase in activity (Figure 1a2,a3). In this example, individuals that do not show a change in activity after elevated CO_2_ exposure may be considered tolerant. Identifying tolerant individuals and their associated behaviours is a critical step in predicting how species will respond and adapt to future ocean acidification conditions.

**FIGURE 1:**
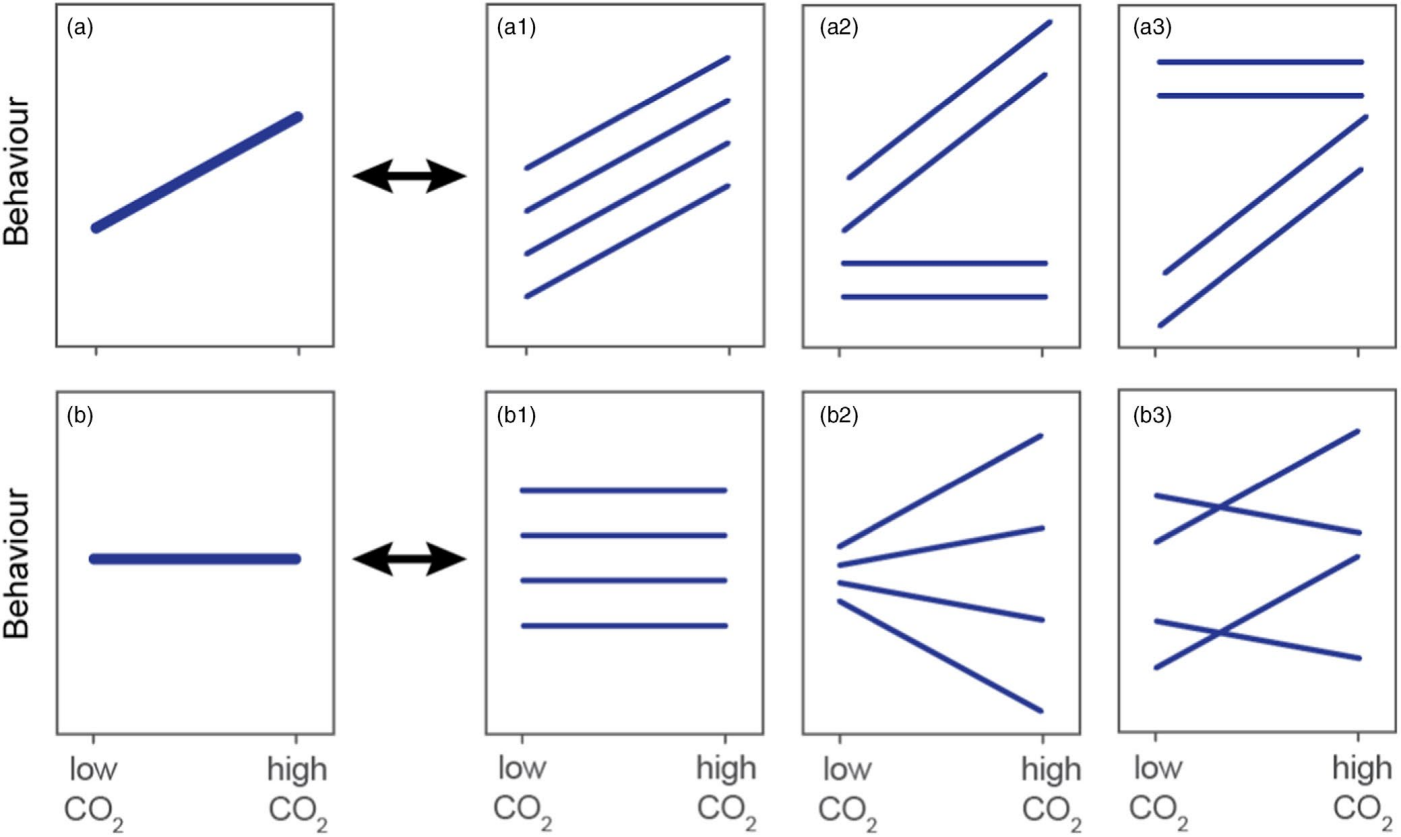
Examples of hypothetical behavioural reaction norms from exposure to high CO_2_. On average, individuals might exhibit either (a) an increase or (b) no change in a given behavioural trait (e.g. activity and anxiety). Different combinations of individual‐level responses can underpin population‐level patterns, where individuals might or might not respond similarly to high CO_2_ exposure. A population‐level increase in behaviour can result from (a1) all individuals increasing their behaviour or (a2, a3) some individuals exhibiting a sharp increase in behaviour and others, no change in behaviour. A lack of population‐level change in behaviour can result from: (b1) the behaviour of all individuals remaining unchanged; (b2) differences in the behaviour of individuals accentuating; or (b3) no consistent changes in behaviour across individuals.

To address several of the knowledge gaps highlighted above, we studied individual‐level behavioural differences and reaction norms in a coral reef damselfish, the Ambon damselfish, *Pomacentrus amboinensis*, at current day, ambient CO_2_ conditions and after 4–6 days at elevated CO_2_ conditions approximating end‐of‐century levels of ocean acidification. We also performed a live predation experiment with the same individuals and the predatory dot‐head rock cod (*Cephalopholis microprion*, hereafter rock cod) to explicitly test the effect of ocean acidification on fitness while considering the influence of individual behavioural differences on the outcome of predator–prey interactions. The Ambon damselfish is common and abundant on many shallow reefs in the Indo‐Pacific. It is planktivorous, diurnal, and commonly preyed upon by mesopredators, including rock cod (Vail & McCormick, 2011). *C. microprion* and *P. amboinensis* have been used as predator and prey, respectively, in previous predation studies (Holmes & McCormick, 2010; Vail & McCormick, 2011).

Our study is the first to explicitly test how individual‐level behavioural variation affects resilience and susceptibility to ocean acidification while incorporating a direct measure of the fitness consequences of any CO_2_‐induced behavioural changes. The approaches used here enable us to address key outstanding questions in this field of research: (1) Are individual‐level behavioural differences in Ambon damselfish repeatable? (2) How does ocean acidification affect population‐ and individual‐level differences in behaviour? (3) Do effects of ocean acidification on behaviour lead to higher predation rates? and (4) Do behavioural traits predict susceptibility to predation? We hypothesized that the repeatability of the behaviours we examined would depend on CO_2_ treatment and that short‐term exposure to elevated CO_2_ would induce behavioural differences compared with individuals in the ambient CO_2_ treatment at both the population and individual levels. We also hypothesized that behavioural traits and short‐term CO_2_ exposure would impact survival in a predation trial with bolder fish acclimated to elevated CO_2_ being the most likely to be eaten quickly.

## 2 MATERIALS AND METHODS

### 2.1 Fish collection and husbandry

All experiments were conducted between 12 January and 1 February 2016. Post‐settlement juvenile *P. amboinensis* were collected by SCUBA divers on 12–13 January from lagoon reefs at Lizard Island on the northern Great Barrier Reef, Australia (14°40′ S; 145° 28′ E) using hand nets and a barrier net (10 mm stretch monofilament). Fish were transported within 90 min of capture to the aquarium facilities at the Lizard Island Research Station (LIRS) in 20 L buckets containing seawater aerated with battery‐operated pumps. At LIRS, the fish were placed in 30 L holding tanks receiving seawater at ~750 mL min^−1^ from one of two flow‐through 32‐L header tanks (receiving seawater pumped directly from the reef) diffused with ambient air. At 24–48 h post‐capture, fish were weighed (Mettler Toledo PL602‐S, d = 0.01 g) and measured for standard length in a water‐filled plastic bag to the nearest 0.1 mm with digital callipers. Seventy‐four *P. amboinensis* (33.1 ± 2.5 mm; 1.43 ± 0.35 g; mean ± SD) were then randomly placed individually in 1‐L flow‐through plastic aquaria (17 × 12 × 7 cm, L × W × H), determined using the randomization tool in Excel 2013 (numbers were randomly generated between 1 and 74 without replacement). These aquaria were supplied with seawater pumped directly from the reef to four header tanks (32 L), and from there diffused to the 74 aquaria (i.e. 18–19 holding aquaria per header tank). The header tanks received seawater at ~750 mL min^−1^, and each 1‐L holding aquaria received seawater at ~40 mL min^−1^. Each aquarium contained a 4 cm long white PVC pipe for shelter. The outer sides of each plastic aquarium were covered in black tape to prevent visual interaction and disturbance. Fish were fed daily with 0.5 mL of a commercial fish flake‐saltwater slurry (TetraMin Tropical Flakes, Tetra, Blacksburg, VA). All animals fed readily by day three post‐capture. Fish were habituated in their aquaria for 3 days prior to the start of the experiment. Seawater temperature in the aquaria was 29.5 ± 1°C. Behavioural assessments for Experiment 1 (below) were performed on each individual twice under ambient CO_2_ conditions, then half the fish were acclimated to elevated CO_2_ for 4–6 days and half were maintained at ambient CO_2_ conditions for the same length of time before behavioural assessments were performed another two times on all individuals. Predation experiments for Experiment 2 (below) were conducted 1–4 days following the last (fourth) behavioural trial.

### 2.2 Experiment 1: Behavioural trials before and after CO_2_ exposure

We examined behavioural differences among *P. amboinensis* using a novel environment assay. This assay is a modified version of the open‐field test in which an individual is introduced into an unfamiliar environment, where the environment also included a novel object (Carter et al., 2013; Roche et al., 2016). Coral reef fishes regularly encounter novel situations and organisms (e.g. unfamiliar predators, substrates and competitors) and must rapidly evaluate potential risks and rewards. Consequently, responses to novel stimuli are ecologically meaningful proxies for risk assessment and exploratory behaviour in reef fishes. Novel environment assays are particularly relevant for Ambon damselfish, which experience predation pressure from a range of mesopredators with different predatory strategies (i.e. ambush and pursuit) as well as inter‐ and intraspecific competition for refuges in the coral matrix. Behavioural assays using novel environments and objects have been used to assess traits, such as boldness and neophobia in coral reef fishes, including damselfishes (e.g. Bowers et al., 2024; White et al., 2013). Each assay lasted 25 min and was repeated twice (2 days between trials) on all fish at ambient CO_2_ (458 ± 17.9 μatm; mean ± standard deviation) and then two more times (2 days between trials) after half the fish were acclimated to elevated CO_2_ and the other half were maintained at ambient CO_2_ (see CO_2_ exposure below and Figure S1). All trials were conducted between 9:00 AM and 04:00 PM, and the time of day was randomized across replicates to control for diurnal patterns in activity. Each trial was filmed (top view) with a digital camera (GoPro Hero 3+ black; GoPro, San Mateo, USA). The stock lens of the camera was replaced with a commercially available lens to avoid image distortion (4.14 mm f/3.0 86° HFOV 5MP GP41430; Peau Productions Inc., San Diego, USA). We presented a note with the fish ID and trial number at the start of each video (Clark et al., 2016). Fish were transferred from their individual aquaria to the experimental arena using their water‐filled shelter (the experimenter blocked both ends of the PVC shelter with the palm of their hands). Fish either entered their shelter when the experimenter approached their aquaria or were carefully placed into the shelter by the experimenter. This procedure minimized air exposure and reduced handling stress prior to introduction into the experimental arena. In instances where a fish was accidentally air exposed, we noted the handling time to account for differences in handling stress prior to the start of the experiment.

The four experimental arenas (38 × 28 × 30 cm, L × W × H) had white, opaque sides and were devoid of structure other than a novel object affixed in the centre. Four different novel objects were used (one object was used per arena at any given trial), each measuring approximately 2 cm in diameter and between 2 and 8 cm in height. The objects differed in shape, height, and colour to enhance the novelty of the stimuli across trials. The object was changed when the assay was repeated, and the order of objects was randomized between fish and trials such that individual fish were presented with each object only once. The experimenter positioned the shelter and the fish at a fixed, pre‐determined location in the arena and exited the room for the duration of the trial to minimize disturbance. The fish was then free to exit its shelter and explore its surroundings over the 25‐min duration of the trial. Water height (seawater pumped directly from the reef) was maintained at 7 cm, and the arena was emptied and rinsed with water between each trial. At the end of each trial, the fish was returned to its individual aquarium with its shelter.

To avoid observer bias, we extracted behavioural data from the videos using the automated tracking software ViewPoint (ZebraLab, Lyon, France). For each trial, we recorded five behavioural measurements: (1) emergence time (the time at which the fish's entire body first exited the shelter in seconds); (2) activity level (the distance covered during the trial [25 min] after emergence in mm per minute), (3) sheltering (the time spent in the vicinity of the shelter [within 3 cm; approximately 1 body length; BL] in seconds); (4) thigmotaxis (the time spent within 3 cm [approximately 1 BL] of the arena walls in seconds); and (5) novel object inspection (time spent within 5 cm i.e. <2 BL, of the novel object) in seconds.

#### 2.2.1 CO_2_ exposure

Once all individuals had repeated the behavioural assay twice under ambient CO_2_, half of the fish (*n* = 37) were maintained in ambient water at present‐day *p*CO_2_ (ambient; 458 ± 17.9 μatm; mean ± SD; min: 450 μatm, max = 490 μatm based on 55 measurements) and the other half (*n* = 37) were exposed to end‐of‐century *p*CO_2_ levels (elevated; 1104 ± 175 μatm; mean ± SD, min: 660 μatm, max = 1490 μatm based on 55 measurements) for 4–6 days. This exposure time was chosen to be consistent with studies reporting altered behaviour in coral reef fishes and reports that longer exposure durations (10 or 25 days) do not alter the response any further (e.g. Allan et al., 2013; Munday et al., 2010, 2013). Control aquaria received seawater from one of two flow‐through 32‐L header tanks diffused with ambient air and filled at ~750 mL min^−1^ from seawater pumped from the reef (i.e. 18–19 holding aquaria per control header tank, flow‐through control seawater in each 1‐L control holding aquaria: ~40 mL min^−1^). CO_2_‐treatment aquaria received water from one of two additional aerated header tanks (32‐L, flow‐through filled at ~750 mL min^−1^, i.e. 18–19 holding aquaria per CO_2_ header tank, flow‐through CO_2_ seawater in each 1‐L CO_2_ holding aquaria: ~40 mL min^−1^) in which the *p*CO_2_ level was gradually increased over 24 h using pH stat computers (Aqua Medic GmbH, Bissendorf, Germany) connected to solenoid valves regulating administration of 100% CO_2_. We monitored *p*CO_2_ in the header tanks and holding tanks multiple times daily using a handheld CO_2_ meter (Vaisala GMT 222, Finland) connected to an aspiration pump (Vaisala GM 70, Finland) and a submerged gas‐permeable PFTE probe (Qubit Systems, Kingston, Canada) following established protocols (Hari et al., 2008; Jutfelt & Hedgärde, 2013). The Vaisala CO_2_ meter was factory calibrated prior to experiments. The experimental design and CO_2_‐dosing system thus followed best practices for ocean acidification research (Cornwall & Hurd, 2015; Riebesell et al., 2011).

### 2.3 Experiment 2: Predation trials

We collected 22 rock cod (*C. microprion*) between 12 and 17 January 2016 from lagoon reefs nearby LIRS using hook‐and‐line. They were transported to the aquarium facility in 20‐L aerated buckets within 2 h of capture and housed in large (~300 L) flow‐through aquaria. We selected 14 individuals that began feeding within 12 h of capture and moved them to individual 32 L white opaque plastic aquaria (38 × 28 × 30 cm, L × W × H). Each aquarium contained a 12.5 cm long PVC pipe (55 mm diameter) providing shelter for the rock cod at one end of the tank. At the other end, we positioned six pieces of half PVC pipe (55 mm diameter) glued together to create a three‐dimensional refuge for *P. amboinensis*, which was inaccessible to *C. microprion*. This allowed us to standardize the refuge size and location across all aquaria. The predator shelter and prey refuge were affixed to the bottom of the tank with silicone. This design intentionally lowered the probability of prey escaping the predator compared with the natural habitat because predation is a rare event that we wanted to observe in our experiment. To habituate rock cod in these aquaria, we fed them pieces of cut, defrosted pilchard during 4 days prior to the experiments. During this time, seven rock cod were kept in present‐day control water (508 ± 48.4 μatm; mean ± SD) and seven were exposed to end‐of‐century *p*CO_2_ levels (1229 ± 252 μatm; mean ± SD) with seawater temperature at approximately 29.5 ± 1°C. CO_2_ dosing and measurements followed the procedures as detailed in the ‘*CO*_*2*_ *exposure*’ section above. Two header tanks were used for the control fish and four for the CO_2_ fish.

Predator trials commenced when Experiment 1 had been completed (January 28–February 1). Twenty minutes before the onset of a predation trial, we introduced an opaque partition in the middle of an aquarium containing a rock cod to restrict *C. microprion* to the area of the tank containing its shelter. One *P. amboinensis* from Experiment 1 was introduced to the other half of the tank, using its PVC shelter as in Experiment 1, and allowed a 20‐min exploration period. We then lifted the partition and allowed the predator and prey to interact for 60 min. We filmed interactions with the same cameras as in Experiment 1 mounted above the aquarium (GoPro Hero 3+ black fitted with a commercial lens). Prey that were not consumed within 60 min were returned to their holding aquarium. Control and CO_2_‐treated *P. amboinensis* were paired with a predator receiving the same experimental treatment using stratified randomization so that each predator received approximately the same number of prey, which spanned a range of behavioural scores. Each predator was tested between five and six times with different prey individuals. Each predator was tested only once in a given day and all predators were fed one piece of pilchard daily in the evening.

### 2.4 Statistical analysis

#### 2.4.1 Experiment 1: Behavioural trials before and after CO_2_ exposure

We checked the distributions of each of the five behavioural measurements (Figure S2) and determined appropriate transformations to comply with the normality assumption of model residuals. We used the R package *lme4* (Bates et al., 2015) to fit five general linear mixed‐effects models (LMM), one for each behavioural measurement. We specified the following fixed effects in each model: treatment (binary variable: ambient vs. elevated CO_2_); trial number (ordinal variable from 1 to 4 with Trials 3 and 4 corresponding to elevated CO_2_ exposure for the treatment group); the interaction between treatment and trial number; and the standard length of fish (continuous variable). The random effects for these models included trial number as random slopes and fish identity as random intercepts. Activity level and thigmotaxis (time spent close to the wall) were square‐root‐transformed; latency to approach a novel object was log‐transformed after adding 0.5; time to emergence from the shelter was log‐transformed; time spent in shelter was not transformed (Figure S2).

We used the *brms* package (Bürkner, 2017) to fit double‐hierarchical general linear mixed‐effects model (DHGLM), one for each of the five behavioural measurements. A DHGLMs comprises two parts, allowing variation in means and variation in variances to be modelled within a single framework (Cleasby et al., 2015; O'Dea et al., 2022). The mean component of the DGHLMs was identical to the LMMs above, and the variation component included the fixed‐effects treatment, trial, and their interaction, as well as the random effect fish identity. The DGHLM allowed us to investigate the effect of CO_2_ treatment on both mean and residual variance (or within‐individual variance), and to compute an estimate of population‐wide variation in predictability (CV_P_, the coefficient of variation in predictability; Cleasby et al., 2015). Predictability refers to the level of variability in an individual's behaviour in a given environment (i.e. within‐individual variation in behaviour), in contrast to repeatability, which is a measure of the consistency in how individuals differ in their average phenotypes (i.e. between‐individual variation in behaviour; see below). CV_P_ represents standardized variation in within‐individual variance among individuals and can serve as a standardized effect size for comparisons across traits, groups or studies.

We used the *rptR* package (Stoffel et al., 2017) with 10,000 bootstrapping iterations to calculate three estimates of repeatability (*R*) for each of the five behavioural measurements: one for all fish, irrespective of treatment group (*N* = 73); one for fish in the ambient CO_2_ treatment group (*N* = 37); and one for fish in the elevated CO_2_ treatment group (*N* = 36). In total, we computed 15 *R* estimates. *R* ranges between 0 and 1 and is the proportion of the total phenotypic variance attributable to differences between individuals; it provides a standardized measure of the consistency of phenotypes across time or contexts (Nakagawa & Schielzeth, 2010; Roche et al., 2016). We note that *R* estimates can be obtained from DGHLMs, but these models could not provide separate estimates for the ambient and elevated CO_2_ treatment groups in our study (see Cleasby et al., 2015). The *rptR* package uses mixed models from the *lme4* package to obtain the repeatability of behavioural measurements; we obtained ‘adjusted’ *R* estimates by fitting trial number as a fixed effect to control for time‐related changes in behaviour (Mitchell et al., 2019). We tested whether *R* differed between the ambient and elevated CO_2_ treatment groups for each behavioural measurement by calculating contrasts between the two groups (five contrasts in total). When the 95% confidence interval (CI) for a given contrast excluded zero, we considered the two *R* estimates to be statistically different.

#### 2.4.2 Experiment 2: Predation trials

We examined differences in the survival of elevated CO_2_‐exposed (*N* = 34) and ambient CO_2_ (*N* = 34) Ambon damsel in predation trials with a Cox Proportional Hazards model, using the *survival* package (Therneau, 2020). Five Ambon damsel used in *Experiment 1* were excluded from this analysis due to issues during the predation trials (i.e. camera malfunction, manipulation error). Seven explanatory variables were included in the model: CO_2_ treatment, prey body size, and the mean of each of the five behavioural measurements recorded four times per Ambon damsel in *Experiment 1*. Predator identity was specified as a cluster (i.e. random) variable to account for repeated measurements. We examined the assumption of proportional hazards using cox.zph() in the package *survival* and *ggcoxdiagnostics*() in the package *survminer* (Therneau, 2020).

We examined differences in the number of predatory attacks resulting in capture for fish in the elevated CO_2_ (*N* = 30) and ambient CO_2_ (*N* = 28) groups with a generalized linear mixed‐effects model (GLMM), specifying a Poisson error distribution. Fixed and random effects in the model were the same as in the survival analysis. Marginal (*R* ^2^_GLMM(m)_) and conditional (*R* ^2^_GLMM(c)_) R‐squared values were computed with *r.squaredGLMM*() in the package *MuMIn* (Barton, 2016). We examined model assumptions with a qqplot of residuals and other diagnostic plots of residuals using *simulateResiduals*() in the package *DHARMa* (Hartig, 2018).

The assumptions of both models were met although the was an indication overdispersion and slight quantile deviations for the GLMM (Dispersion test *p* = 0.040). All analyses were done in R 4.2.2 (R Core Team, 2023). We used the packages *ggplot2* (Wickham, 2016), *tidybayes* (Kay, 2024), *emmeans* (Lenth, 2025) and *survminer* (Therneau, 2020) for data visualization.

## 3 RESULTS

### 3.1 Experiment 1: Behavioural trials before and after CO_2_ exposure

Short‐term exposure to projected end‐of‐century CO_2_‐induced acidification had no effect on any of the five behaviours measured in *P. amboinensis*, both in terms of group means (i.e. population‐level; Figure 2, Table 1) and residual (i.e. within‐individual) variance (Figure 3, Table 1). Thigmotaxis decreased, and time spent near the novel object increased with trial number for fish in both the ambient CO_2_ and elevated CO_2_ groups, indicating habituation to the experimental setup and/or procedure in both treatment groups (Figure 2, Table 1). No such population‐level trends were observed for emergence time, activity level or time spent sheltering (Figure 2, Table 1). Intra‐individual variability decreased with trial number for all five behaviours for fish in both the ambient CO_2_ and elevated CO_2_ groups (Figure 3), indicating some degree of habituation to the setup/manipulation.

**FIGURE 2:**
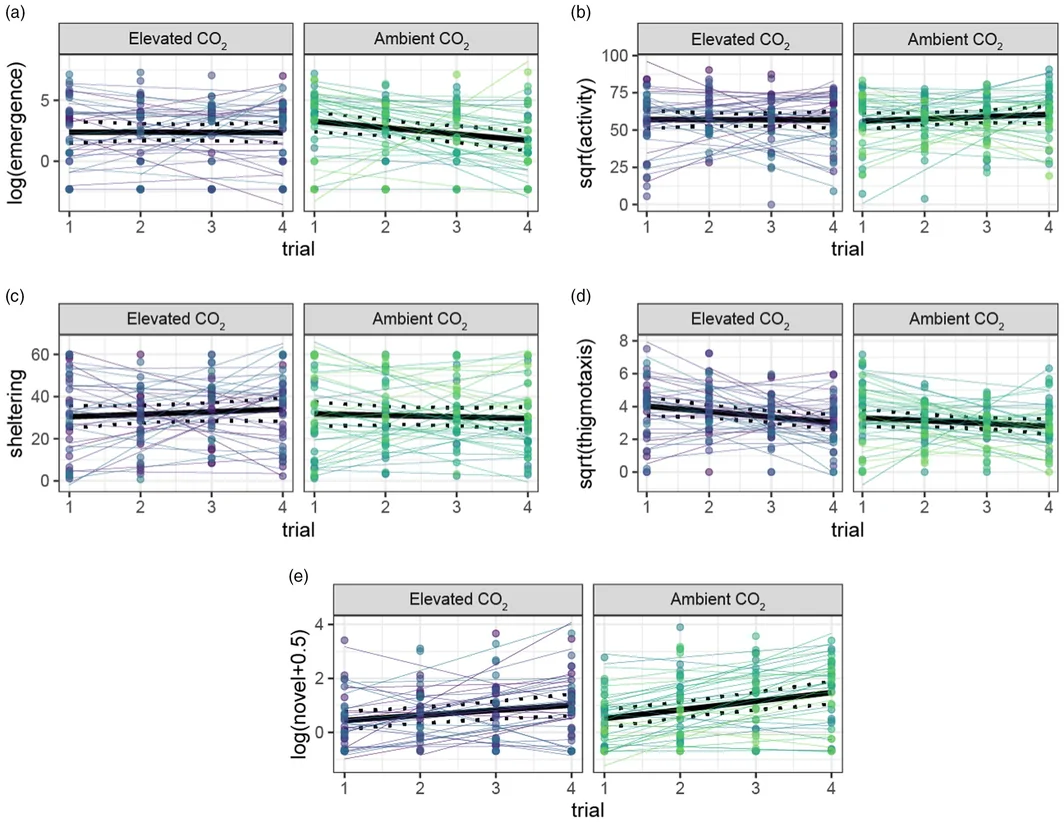
Reaction norm plots of five behavioural measurements recorded across four trials for Ambon damselfish (*P. amboinensis*) exposed to present‐day levels of dissolved CO_2_ (Ambient CO_2_; *n* = 36) and projected end‐of‐century CO_2_‐induced aquatic acidification (Elevated CO_2_, *n* = 37): (a) time to emergence from the shelter, (b) activity, (c) time sheltering, (d) thigmotaxis and (e) inspection of a novel object. Fish in the Ambient CO_2_ group were exposed to present‐day CO_2_ levels in all four trials, whereas fish in the Elevated CO_2_ group were exposed to present‐day CO_2_ levels in Trials 1–2 and projected end‐of‐century CO_2_ levels in Trials 3–4. Data points and coloured lines represent individual fish; the black line is the population‐level (i.e. mean) response with the 95% CI credible interval indicated by dotted lines. Non‐transformed data are shown in Figure S3.

**TABLE 1:** Estimates and 95% confidence intervals (CI) for predictors included in five double‐hierarchical linear mixed‐effects models (DHGLMs) assessing how end‐of‐century CO_2_‐induced aquatic acidification affects five behavioural measurements in the Ambon damselfish (*Pomacentrus amboinensis*): Activity level (Activity), emergence time (Emergence), time spent inspecting a novel object (Novel), time sheltering (Sheltering), time close to the arena walls (Thigmotaxis).

Predictor	Activity (95% CI)	Emergence (95% CI)	Novel (95% CI)	Sheltering (95% CI)	Thigmotaxis (95% CI)
Intercept	57.74 (53.40, 61.90)	2.45 (1.74, 3.18)	0.70 (0.39, 1.02)	32.17 (27.69, 36.69)	3.55 (3.17, 3.92)
sigma_Intercept	2.37 (2.10, 2.63)	0.73 (0.50, 0.95)	−0.44 (−0.66, −0.22)	2.41 (2.19, 2.62)	0.23 (0.01, 0.43)
1. groupcontrol	1.05 (−4.65, 7.00)	0.09 (–0.92, 1.08)	0.27 (−0.17, 0.73)	−1.37 (−7.76, 4.90)	−0.48 (−1.01, 0.04)
2. trial	−0.96 (−6.60, 4.83)	−0.13 (−0.80, 0.56)	**0.51** (0.18, 0.83)	2.53 (−3.08, 8.17)	**−0.77** (−1.31, −0.21)
3. SL	0.24 (−5.60, 6.13)	0.6 (−0.40, 1.61)	0.19 (−0.24, 0.63)	−5.09 (−11.48, 1.46)	0.16 (−0.36, 0.66)
4. groupcontrol:trial	3.42 (−4.51, 11.30)	**−1.64** (−2.54, −0.70)	0.24 (−0.21, 0.69)	−4.5 (−12.29, 3.09)	0.30 (−0.48, 1.07)
5. sigma_groupcontrol	0.01 (−0.34, 0.37)	−0.13 (−0.43, 0.19)	0.06 (−0.24, 0.36)	−0.06 (−0.35, 0.22)	−0.12 (−0.39, 0.15)
6. sigma_trial	**−0.56** (−1.01, −0.11)	−0.2 (−0.58, 0.15)	−0.24 (−0.69, 0.23)	−0.22 (−0.66, 0.26)	−0.24 (−0.64, 0.16)
7. sigma_groupcontrol:trial	0.15 (−0.48, 0.80)	0.27 (−0.25, 0.82)	0.13 (−0.5, 0.78)	−0.34 (−1.01, 0.28)	−0.23 (−0.78, 0.31)

*Note*: Predictors 1–4 relate to the mean component of the DHGLMs and Predictors 5–7 relate to the dispersion component (i.e. residual variances for individuals). Estimates in bold have a 95% credible interval non‐overlapping zero.

**FIGURE 3:**
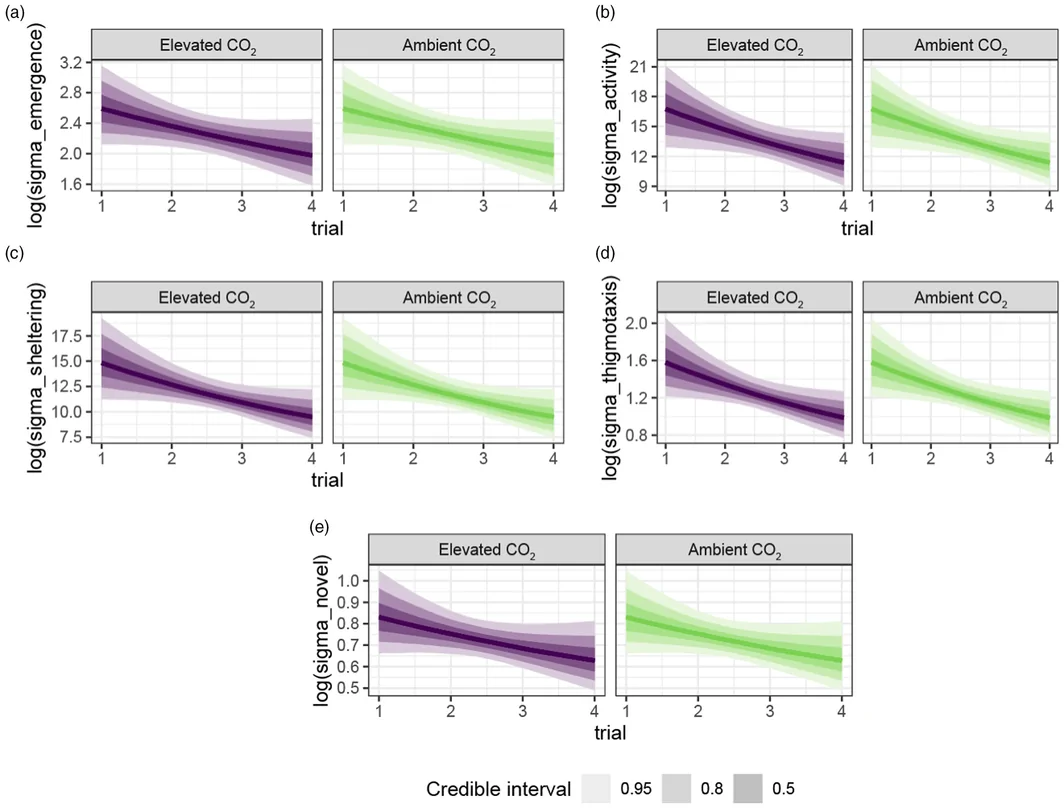
Intra‐individual variation (residual variance for individual fish) in five behavioural measurements across four trials in Ambon damselfish (*P. amboinensis*) exposed to present‐day levels of dissolved CO_2_ (Ambient CO_2_, *n* = 36) and projected end‐of‐century CO_2_‐induced aquatic acidification (Elevated CO_2_, *n* = 37): (a) time to emergence from the shelter, (b) activity, (c) time sheltering, (d) thigmotaxis and (e) inspection of a novel object. Fish in the Ambient CO_2_ group were exposed to present‐day CO_2_ levels in all four trials, whereas fish in the Elevated CO_2_ group were exposed to present‐day CO_2_ levels in Trials 1 and 2, and projected end‐of‐century CO_2_ levels in Trials 3 and 4.

All behaviours were moderately and significantly repeatable, with no marked differences in repeatability estimates between CO_2_ groups (Table 2). There was substantial variability in predictability (i.e. within‐individual variance) for all five behavioural measurements for both groups (CV_p_; see Table 1).

**TABLE 2:** Estimates of repeatability (*R*) and variation in predictability (CVp) for five behavioural traits measured experimentally four times in the Ambon damselfish, *Pomacentrus amboinensis*.

Behaviour	Overall *R* (95% CI)	Control *R* (95% CI)	Treatment *R* (95% CI)	Contrast (95% CI)	CVp (95% CI)
Activity	0.278 (0.149, 0.402)	0.255 (0.068, 0.427)	0.303 (0.113, 0.472)	0.046 (−0.211, 0.302)	0.805 (0.625, 0.997)
Emergence	0.298 (0.169, 0.424)	0.317 (0.124, 0.488)	0.303 (0.118, 0.478)	−0.015 (−0.269, 0.247)	0.754 (0.533, 0.967)
Novel object	0.497 (0.363, 0.605)	0.568 (0.390, 0.703)	0.405 (0.210, 0.571)	−0.162 (−0.403, 0.082)	0.636 (0.324, 0.856)
Shelter	0.381 (0.247, 0.502)	0.427 (0.230, 0.588)	0.340 (0.147, 0.509)	−0.085 (−0.335, 0.174)	0.545 (0.202, 0.775)
Thigmotaxis	0.245 (0.118, 0.368)	0.243 (0.066, 0.413)	0.225 (0.043, 0.398)	−0.020 (−0.271, 0.229)	0.568 (0.175, 0.805)

*Note*: Behaviour was assessed in all fish under ambient CO_2_ conditions twice, and thereafter twice more after having exposed half of the fish to elevated CO_2_. The difference in *R* between the ambient CO_2_ group (control) and elevated CO_2_ treatment group for each behaviour is presented as a contrast (Contrast). 95% confidence intervals are indicated in parentheses. The behaviours measured were activity level (Activity), emergence time (Emergence), time spent inspecting a novel object (Novel), time sheltering (Sheltering), and time close to the arena walls (Thigmotaxis).

### 3.2 Experiment 2: Predation trials

The probability of survival in a predation trial was similar for Ambon damsel in the elevated CO_2_ and ambient groups (Figure 4), with fish in both treatments having a similar hazard of death (ambient CO_2_ treatment hazard ratio relative to elevated CO_2_ treatment group = 0.88, 95% CI = 0.43–1.78, *p* = 0.719; Table 3). While body size, emergence time, activity level, time sheltering, and thigmotaxis were unrelated to survival probability (Table 3), Ambon damsel that spent a greater amount of time inspecting a novel object had a lower probability of survival (hazard ratio = 2.66, 95% CI = 1.36–5.20, *p* = 0.004; Table 3).

**FIGURE 4:**
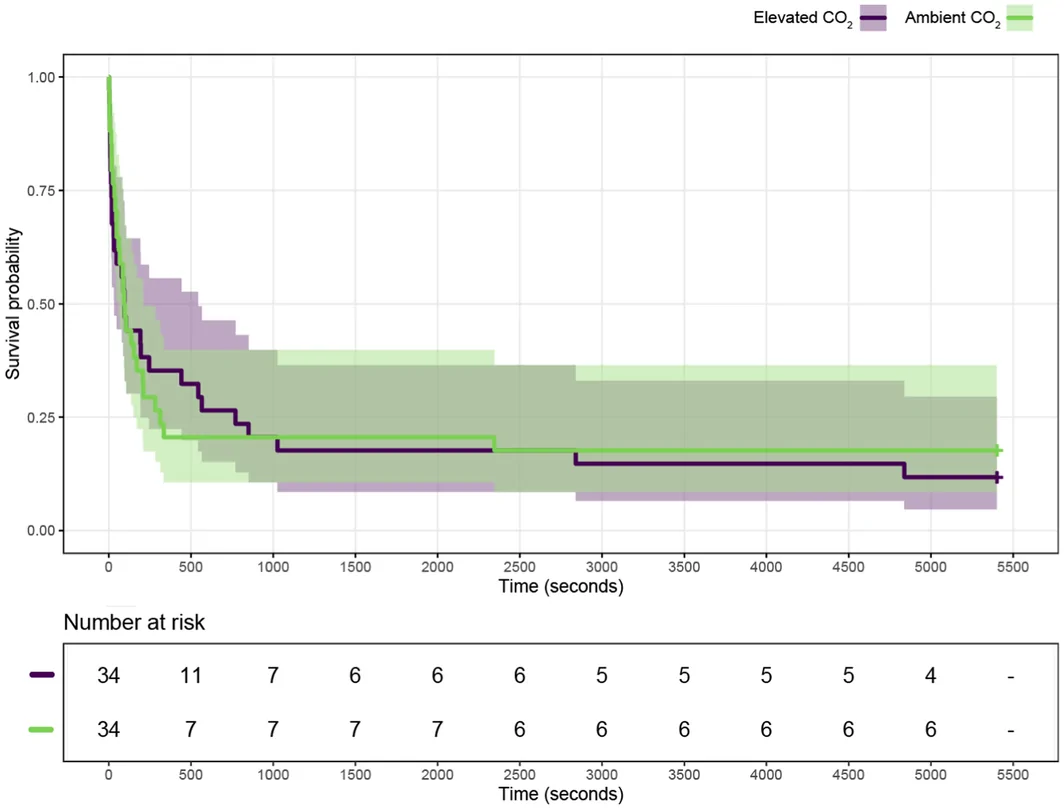
Kaplan–Meier survival curves for Ambon damselfish (*P. amboinensis*) exposed to present‐day levels of dissolved CO_2_ (Ambient CO_2_, *n* = 34) and projected end‐of‐century CO_2_‐induced aquatic acidification (Elevated CO_2_, *n* = 34). Fish were subjected to a 90 min predation trial involving a predator, the rock cod *Cephalopholis microprion*, exposed to the same conditions of water chemistry.

**TABLE 3:** Hazard ratios (HR) for fixed‐effects predictors in a Cox Proportional Hazards model examining differences in survival probability for CO_2_‐exposed (*N* = 34) and control (*N* = 34) Ambon damsel (*Pomacentrus amboinensis*) (prey).

Predictor	HR	95% CI	*Z*	*p*
Prey standard length	1.11	0.87, 1.42	0.833	0.405
Treatment_control	0.88	0.43, 1.78	−0.360	0.719
Emergence time	1.11	0.84, 1.47	0.746	0.456
Activity level	1.36	0.87, 2.14	1.349	0.177
Time sheltering	2.99	0.90, 9.96	1.785	0.074
Thigmotaxis	1.60	0.69, 3.71	1.084	0.278
Novel object	2.66	1.36, 5.20	2.871	0.004

*Note*: A hazard ratio lower than 1 indicates reduced hazard of death whereas a hazard ratio greater than 1 indicates an increased hazard of death. 95% confidence intervals, *Z* scores, and *p*‐values are indicated. The predator (*Cephalopholis microprion*) was exposed to the same conditions of water chemistry as the prey.

Rock cod often failed to capture their prey upon striking: only 6 of 58 Ambon damsel that were ultimately eaten were captured on the first strike, with several evading their predator more than 10 times (Figure S4). Ten Ambon damsel were not captured during the 60‐min trial (Figure 4). The number of predatory attacks leading to capture was similar for fish in the elevated CO_2_ (mean ± SD: 5.96 ± 3.18) and ambient CO_2_ groups (mean ± SD: 5.39 ± 3.86) (GLMM estimate ± SE: −0.040 ± 0.182, *p* = 0.827; Table S1, Figure S4). Predators required more attacks to capture large Ambon damsel (estimate ± SE: 0.147 ± 0.067, *p* = 0.027) as well as Ambon damsel that spent more time near a shelter (estimate ± SE: 0.490 ± 0.243, *p* = 0.044) and in the vicinity of the novel object during the repeated behavioural trials (estimate ± SE: 0.386 ± 0.137, *p* = 0.005) (Table S1; marginal *R* ^2^_GLMM_ = 0.19; conditional *R* ^2^_GLMM_ = 0.35).

## 4 DISCUSSION

Studies on the impacts of end‐of‐century ocean acidification on coral reef fish behaviour are largely based on comparisons between treatment means rather than individual‐level effects. Although it has been argued that some individuals are potentially more tolerant to elevated CO_2_ exposure than others (Esbaugh, 2018; Tresguerres & Hamilton, 2017), the extent to which individual behaviour affects responses to ocean acidification has never been tested.

We investigated the repeatability, individual‐ and population‐level differences in five commonly measured behaviours as well as survival outcomes during a predation trial in the context of ocean acidification. All individuals habituated to the experimental setup as evidenced by decreased intra‐individual variability over the course of the four behavioural trials (Figure 3). Habituation to experimental arenas is common when repeatedly measuring behaviour, especially in laboratory settings when the timing between assays is short (Blumstein, 2016; Martin & Réale, 2008). Nevertheless, we found that all five behaviours measured were significantly repeatable in both treatments, indicating that our assays measured consistent behavioural differences in Ambon damsels. Our repeatability estimates (0.278–0.497; Table 2) are relatively high considering that when measured in the laboratory and on ectotherms, estimates tend to be lower than when measured in the field and/or on endotherms (Bell et al., 2009). Contrary to widespread assumptions, this behavioural consistency was not affected by CO_2_ treatment: both control and elevated CO_2_ groups had similar estimates of repeatability in all behaviours, and population reaction norms followed similar trends in both groups even after CO_2_ exposure (scenario B1 in Figure 1). This finding suggests that individual behavioural traits are not differentially impacted by several days of CO_2_ exposure, providing evidence refuting the suggestion that some individual phenotypes are more tolerant or susceptible to elevated CO_2_ exposure than others.

When population‐level differences in behaviours were considered, exposure to projected end‐of‐century CO_2_ had no effect on any of the five behaviours measured in Ambon damselfish. Our results are in line with Clark et al. (2020) suggesting that short‐term CO_2_ exposure does not meaningfully alter coral reef fish behaviour. This result contrasts with earlier literature showing large effects of short‐term CO_2_ exposure on many aspects of fish behaviour (reviewed in Briffa et al., 2012; Clark et al., 2024; Clements & Hunt, 2015; Clements et al., 2022; Sundin, 2023). A lack of a CO_2_ treatment effect is perhaps not surprising since fishes have a well‐developed acid–base regulatory system, which allows them to maintain tissue pH under acidified conditions (Ishimatsu et al., 2005). In fact, more than a century of research has shown that fish physiology is generally tolerant to elevated CO_2_ levels, including those that exceed climate change projections (Clark et al., 2024). That behavioural responses are similarly resilient is consistent with this idea, and there is growing empirical evidence of a decline effect in results testing for behavioural differences between control individuals and individuals exposed to ocean acidification (Clements et al., 2022, 2023). The results from our carefully designed study provide robust evidence that fish behaviour is not altered following short‐term exposure to elevated CO_2_ whether individual or population‐level effects are considered. Although ocean acidification may indeed have negative impacts on some marine life, especially calcifying organisms (Clark et al., 2024; Kroeker et al., 2010), our results highlight the growing agreement around the behavioural resilience of fishes to ocean acidification, as well as the value of transparent and reproducible research practices in achieving consensus around controversial research topics (O'Dea et al., 2021; Roche et al., 2022).

CO_2_ treatment had no effect on the probability of prey fish surviving in a predation trial; Ambon damsel from both treatment groups had a similar hazard of death (Figure 4). The number of predatory attacks leading to capture was also similar between treatment groups (Figure S4). The experimental arena was intentionally designed to increase predator–prey encounter rates relative to those experienced on natural reefs so that predation attempts could be observed within our experimental period. Consequently, the assay was intended to provide a standardized comparison of relative predator evasion between experimental treatments rather than to estimate absolute predation risk in nature. Although escape opportunities for the damselfish in our arena were reduced compared with what they might have access to in the field, individuals frequently evaded repeated attacks and approximately 15% survived the entire trial (Figure 4; Figure S4), indicating that substantial variation in escape performance remained despite the constrained space. These results are consistent with several other studies suggesting that CO_2_ exposure has limited effects on predator–prey interactions in coral reef fishes (Allan et al., 2017; Ferrari et al., 2017; McCormick et al., 2018), refuting early assertions, based on indirect evidence, that ocean acidification dramatically increases prey susceptibility to predation (e.g. Devine & Munday, 2013; Munday et al., 2010). Indeed, Allan et al. (2013) found that when both the predator and prey had been exposed to elevated CO_2_ (as in our study), the capture rate was similar to that of the control group (where both predators and prey were exposed to present‐day CO_2_). Similarly, a study exploring the effect of elevated CO_2_ on predation in freshwater fishes found no effect of CO_2_ treatment on predator success or time to predation (Midway et al., 2017). Our results further highlight the importance of behavioural tests on interacting animals when assessing the impact of anthropogenic stressors such as ocean acidification on complex ecological interactions such as predation.

Although elevated CO_2_ had no impact on survival outcomes during predation trials in our study, we did observe small differences in the survival of Ambon damsel based on individual traits. We found that predators required more attacks to capture large Ambon damsel, which may be due to minor differences in mouth gape requirements of the predators and/or higher absolute burst swimming speeds in larger versus smaller Ambon damsel conspecifics (Taylor & McPhail, 1985). While emergence time, activity level, time spent sheltering, and thigmotaxis behaviours were not related to individual survival, Ambon damsels that spent a greater amount of time inspecting the novel object had a lower probability of survival. This trend was independent of CO_2_ treatment. Our results are consistent with the notion that bolder individuals face higher predation risk than shy individuals (Hulthén et al., 2017). However, as four out of five behaviours tested had no relationship with survival, the role of individual behavioural traits in predicting predation risk should be interpreted with caution. Furthermore, although we predicted higher mortality in bolder fish from the elevated CO_2_ treatment group compared with the control group, the effect of behaviour on survival outcome was independent of CO_2_ exposure. This result runs counter to those of several studies that have linked elevated CO_2_ to increased boldness in fishes (e.g. Ferrari et al., 2011, 2017; Mitchell et al., 2022; Munday et al., 2014). Again, our results align with more recent studies demonstrating negligible effects of ocean acidification on fish behaviour (Clark et al., 2020, 2024; Clements et al., 2022, 2023) and do not support the notion of differences in individual tolerance or susceptibility to ocean acidification within a fish population.

We used elevated CO_2_ exposure durations of 4–6 days in our experiments, which is consistent with studies that remain the most highly cited in the field (see Clements et al., 2022), and comparable with recent studies testing CO_2_‐induced behavioural alterations (e.g. 5–7 days in Hamilton et al., 2023; 10 days in Durant et al., 2023). In the limited number of studies that have examined the topic, longer exposure durations have not been reported to cause more pronounced behaviour differences between treatment groups: Munday et al. (2010) concluded for larval/settlement stages of clownfish and damselfish that behavioural impairments were maximized at 4 days, and Munday et al. (2013) did not report any change in the behavioural impairments of juvenile coral trout (*Plectropomus leopardus*) after 28 days of elevated CO_2_ exposure compared with the impairments already existing after 4 days, suggesting that behavioural impairments do not increase proportionally with exposure time. Clark et al. (2024) analysed existing data for the effects of CO_2_ on chemical cue detection and found no effect of fish age or duration of CO_2_ exposure on the outcomes (see fig. 5.2, table 5.2 in Clark et al., 2024). Thus, it is unlikely that the lack of behavioural differences we observed is solely due to the short duration of CO_2_ exposure used.

## 5 CONCLUSION

Ocean acidification presents a growing threat to marine ecosystems globally because of anthropogenic activities. Concerns for how this change in water chemistry will impact aquatic organisms are warranted and an important area of continued research. Yet, there are limited resources to understand the complexities of ocean acidification impacts on marine life and thus researchers should focus their time and effort on species, life stages, interactions and physiological processes that are most likely to suffer consequences as a result of ocean acidification in order to inform decision making and conservation strategies (Buxton et al., 2021). Based on our results, as well as the growing body of evidence from different species and study systems, we suggest that fish behaviour is not likely to be meaningfully impacted by near‐future ocean acidification levels. Thus, future research should prioritize studying how other aspects of elevated atmospheric CO_2_ impact marine fishes, including the rapid rise in the temperature of the global oceans.

## AUTHOR CONTRIBUTIONS

Dominique G. Roche, Ben Speers‐Roesch, Josefin Sundin, Timothy D. Clark and Sandra A. Binning conceived the ideas and designed methodology. Dominique G. Roche, Ben Speers‐Roesch and Sandra A. Binning collected the data. Timothy D. Clark processed the data. Dominique G. Roche and Shinichi Nakagawa analysed the data. Dominique G. Roche and Sandra A. Binning led the writing of the manuscript with substantial input from Josefin Sundin and Timothy D. Clark. All authors contributed critically to the drafts and gave final approval for publication. Our study brings together authors from a number of different countries, including two scientists based in Australia where the study was carried out.

## CONFLICT OF INTEREST STATEMENT

None to declare.

## ETHICS STATEMENT

Animals were collected and cared for under Marine Parks Permit no. G13/35909.1 issued by the Australian Government's Great Barrier Reef Marine Park Authority. All experiments were approved by the Animal Experimentation Ethics Committee at James Cook University (permit no.: A1924) according to the Australian Code of Practice for the Care and Use of Animals for Scientific Purposes, 7th edition 2007. AI was not used in any part of the study, data analysis or manuscript creation with the exception of abstract translation.

## Supporting information


**Table S1.** Summary table with estimates, standard errors (SE), *Z* and *p*‐values for fixed effects included in a Poisson generalized linear mixed‐effects model examining differences in number of predatory attacks that resulted in the capture of Ambon damsel (*Pomacentrus amboinensis*) in ambient CO_2_ (*N* = 28) and elevated CO_2_ conditions (*N* = 30). The sample size excludes Ambon damsel that were not captured. Predators (*Cephalopholis microprion*) were exposed to the same conditions of water chemistry as their prey. Predator identity was included as a random effect.
**Figure S1.** Graphical representation of the experimental timeline used to examine the effect of exposure to end‐of‐century CO_2_ levels on personality and susceptibility to predation in *Pomacentrus amboinensis*. Fish personality was assessed by repeatedly measuring behaviour in the same fish in a novel environment assay on specific days (orange arrows) during 4 days exposure to present day, ambient, CO_2_ (control, *n* = 74) followed the next day by treatment with either present‐day CO_2_ or elevated CO_2_ for 6 days (*n* = 37 each). At the end of the CO_2_ treatments, each fish was exposed to a predator–prey trial (using *Cephalopholis microprion* as predator) under the same CO_2_ treatment.
**Figure S2.** The distribution of model residuals (m_) after the following transformations: time to emergence from the shelter (emergT) was log‐transformed; activity levels (activity) and thigmotaxis (thigmotaxis) were square‐root‐transformed; time spent sheltering (shelter) was not transformed; and time near a novel (novel) object was log‐transformed after adding 0.5.
**Figure S3.** Reaction norm plots of non‐transformed data for five behavioural measurements recorded across four trials for Ambon damselfish (*P. amboinensis*) exposed to present‐day levels of dissolved CO_2_ (Ambient CO_2_; *n* = 36) and projected end‐of‐century CO_2_‐induced aquatic acidification (Elevated CO_2_, *n* = 37): (A) emergence, (B) activity, (C) time sheltering, (D) thigmotaxis and (E) inspection of a novel object. Fish in the control group were exposed to present‐day CO_2_ levels in all four trials, whereas fish in the CO_2_ group were exposed to present‐day CO_2_ levels in Trials 1–2 and projected end‐of‐century CO_2_ levels in Trials 3–4. Data points and coloured lines represent individual fish; the black line is the population‐level (i.e. mean) response with the 95% CI credible interval indicated by dotted lines.
**Figure S4.** Number of predatory attacks by dot‐head rock cod (*Cephalopholis microprion*) resulting in the capture of Ambon damsel (*Pomacentrus amboinensis*) in ambient CO_2_ (*N* = 28) and elevated CO_2_ (*N* = 30) treatment groups. Ambon damsels that were not captured are not shown.

## Data Availability

The data and analytical code to reproduce the results of this study were shared with the editors and reviewers on submission and are publicly available on the Open Science Framework: https://doi.org/10.17605/OSF.IO/CZVSF (Roche et al., 2026).

## References

[jane70346-bib-0001] Allan, B. J. M. , Domenici, P. , McCormick, M. I. , Watson, S.‐A. , & Munday, P. L. (2013). Elevated CO_2_ affects predator‐prey interactions through altered performance. PLoS One, 8, e58520. 10.1371/journal.pone.0058520 PMC3590170 23484032

[jane70346-bib-0002] Allan, B. J. M. , Domenici, P. , Watson, S. A. , Munday, P. L. , & McCormick, M. I. (2017). Warming has a greater effect than elevated CO_2_ on predator‐prey interactions in coral reef fish. Proceedings of the Royal Society B: Biological Sciences, 284, 20170784. 10.1098/rspb.2017.0784 PMC5489729 28659450

[jane70346-bib-0003] Barton, K. (2016). Package “MuMIn”: Multi‐modal inference. R package version 1.15.6. https://cran.r‐project.org/web/packages/MuMIn/MuMIn.pdf.

[jane70346-bib-0004] Bates, D. , Mächler, M. , Bolker, B. , & Walker, S. (2015). Fitting linear mixed‐effects models using lme4. Journal of Statistical Software, 67, 1–48.

[jane70346-bib-0005] Bell, A. M. , Hankison, S. J. , & Laskowski, K. L. (2009). The repeatability of behaviour: A meta‐analysis. Animal Behaviour, 77, 771–783. 10.1016/j.anbehav.2008.12.022 PMC3972767 24707058

[jane70346-bib-0006] Blumstein, D. T. (2016). Habituation and sensitization: New thoughts about old ideas. Animal Behaviour, 120, 255–262.

[jane70346-bib-0007] Bowers, R. , Burgos, N. , Meshanko, R. , Thaker, S. , Yan, A. , O'Fallon, S. , & Blumstein, D. T. (2024). Does visual or mechanosensory disruption influence risk assessment in coral reef fishes: A preliminary study. Journal of Fish Biology, 105, 1919–1928. 10.1111/jfb.15941 PMC11650934 39305098

[jane70346-bib-0008] Briffa, M. , de la Haye, K. , & Munday, P. L. (2012). High CO_2_ and marine animal behaviour: Potential mechanisms and ecological consequences. Marine Pollution Bulletin, 64, 1519–1528. 10.1016/j.marpolbul.2012.05.032 22749063

[jane70346-bib-0009] Browman, H. I. (2016). Applying organized scepticism to ocean acidification research. ICES Journal of Marine Science, 73, 529–536.

[jane70346-bib-0010] Bürkner, P.‐C. (2017). brms: An R package for Bayesian multilevel models using Stan. Journal of Statistical Software, 80, 1–28.

[jane70346-bib-0011] Buxton, R. T. , Nyboer, E. A. , Pigeon, K. E. , Raby, G. D. , Rytwinski, T. , Gallagher, A. J. , Schuster, R. , Lin, H.‐Y. , Fahrig, L. , Bennett, J. R. , Cooke, S. J. , & Roche, D. G. (2021). Avoiding wasted research resources in conservation science. Conservation Science and Practice, 3, e329.

[jane70346-bib-0012] Carter, A. J. , Feeney, W. E. , Marshall, H. H. , Cowlishaw, G. , & Heinsohn, R. (2013). Animal personality: What are behavioural ecologists measuring? Biological Reviews, 88, 465–475. 10.1111/brv.12007 23253069

[jane70346-bib-0013] Clark, T. D. , Binning, S. A. , Raby, G. D. , Speers‐Roesch, B. , Sundin, J. , Jutfelt, F. , & Roche, D. G. (2016). Scientific misconduct: The elephant in the lab. A Rresponse to Parker et al. Trends in Ecology & Evolution, 31, 899–900. 10.1016/j.tree.2016.09.006 27707511

[jane70346-bib-0014] Clark, T. D. , Clements, J. C. , Jutfelt, F. , & Sundin, J. (2024). Ocean acidification. In B. B. M. Wong & U. Candolin (Eds.), Behavioural responses to a changing world (pp. 72–96). Oxford University Press.

[jane70346-bib-0015] Clark, T. D. , Raby, G. D. , Roche, D. G. , Binning, S. A. , Speers‐Roesch, B. , Jutfelt, F. , & Sundin, J. (2020). Ocean acidification does not impair the behaviour of coral reef fishes. Nature, 577, 370–375. 10.1038/s41586-019-1903-y 31915382

[jane70346-bib-0016] Cleasby, I. R. , Nakagawa, S. , & Schielzeth, H. (2015). Quantifying the predictability of behaviour: Statistical approaches for the study of between‐individual variation in the within‐individual variance. Methods in Ecology and Evolution, 6, 27–37.

[jane70346-bib-0017] Clements, J. C. , & Hunt, H. L. (2015). Marine animal behaviour in a high CO_2_ ocean. Marine Ecology Progress Series, 536, 259–279.

[jane70346-bib-0018] Clements, J. C. , Sundin, J. , Clark, T. D. , & Jutfelt, F. (2022). Meta‐analysis reveals an extreme “decline effect” in the impacts of ocean acidification on fish behavior. PLoS Biology, 20, e3001511. 10.1371/journal.pbio.3001511 PMC8812914 35113875

[jane70346-bib-0019] Clements, J. C. , Sundin, J. , Clark, T. D. , & Jutfelt, F. (2023). Extreme original data yield extreme decline effects. PLoS Biology, 21, e3001996. 10.1371/journal.pbio.3001996 PMC9901777 36745659

[jane70346-bib-0020] Cornwall, C. E. , & Hurd, C. L. (2015). Experimental design in ocean acidification research: Problems and solutions. ICES Journal of Marine Science, 73, 572–581.

[jane70346-bib-0021] Devine, B. M. , & Munday, P. L. (2013). Habitat preferences of coral‐associated fishes are altered by short‐term exposure to elevated CO_2_ . Marine Biology, 160, 1955–1962.

[jane70346-bib-0022] Devine, B. M. , Munday, P. L. , & Jones, G. P. (2012). Homing ability of adult cardinalfish is affected by elevated carbon dioxide. Oecologia, 168, 269–276. 10.1007/s00442-011-2081-2 21800059

[jane70346-bib-0023] Dingemanse, N. J. , & Dochtermann, N. A. (2013). Quantifying individual variation in behaviour: Mixed‐effect modelling approaches. Journal of Animal Ecology, 82, 39–54. 10.1111/1365-2656.12013 23171297

[jane70346-bib-0024] Dingemanse, N. J. , Kazem, A. J. , Réale, D. , & Wright, J. (2010). Behavioural reaction norms: Animal personality meets individual plasticity. Trends in Ecology & Evolution, 25, 81–89. 10.1016/j.tree.2009.07.013 19748700

[jane70346-bib-0025] Dixson, D. L. , Munday, P. L. , & Jones, G. P. (2026). RETRACTION: Ocean acidification disrupts the innate ability of fish to detect predator olfactory cues. Ecology Letters, 29, e70445. 10.1111/ele.70445 PMC13441514 42556795

[jane70346-bib-0026] Draper, A. M. , & Weissburg, M. J. (2019). Impacts of global warming and elevated CO_2_ on sensory behavior in predator‐prey interactions: A review and synthesis. Frontiers in Ecology and Evolution, 7, 72.

[jane70346-bib-0027] Dubois, F. , & Binning, S. A. (2022). Predation and parasitism as determinants of animal personalities. Journal of Animal Ecology, 91, 1918–1928. 10.1111/1365-2656.13781 35856175

[jane70346-bib-0028] Durant, A. , Khodikian, E. , & Porteus, C. S. (2023). Ocean acidification alters foraging behaviour in Dungeness crab through impairment of the olfactory pathway. Global Change Biology, 29, 4126–4139. 10.1111/gcb.16738 37158617

[jane70346-bib-0029] Esbaugh, A. J. (2018). Physiological implications of ocean acidification for marine fish: Emerging patterns and new insights. Journal of Comparative Physiology B, 188, 1–13. 10.1007/s00360-017-1105-6 28547292

[jane70346-bib-0030] Ferrari, M. C. O. , Dixson, D. L. , Munday, P. L. , McCormick, M. I. , Meekan, M. G. , Sih, A. , & Chivers, D. P. (2011). Intrageneric variation in antipredator responses of coral reef fishes affected by ocean acidification: Implications for climate change projections on marine communities. Global Change Biology, 17, 2980–2986.

[jane70346-bib-0031] Ferrari, M. C. O. , McCormick, M. I. , Watson, S.‐A. , Meekan, M. G. , Munday, P. L. , & Chivers, D. P. (2017). Predation in high CO_2_ waters: Prey fish from high‐risk environments are less susceptible to ocean acidification. Integrative and Comparative Biology, 57, 55–62. 10.1093/icb/icx030 28881940

[jane70346-bib-0032] Gradito, M. , Dubois, F. , Noble, D. W. A. , & Binning, S. A. (2024). Double trouble: Host behaviour influences and is influenced by co‐infection with parasites. Animal Behaviour, 215, 31–44.

[jane70346-bib-0033] Hamilton, T. J. , Tresguerres, M. , T. Kwan, G. T. , Szaskiewicz, J. , Franczak, B. , Cyronak, T. , Andersson, A. J. , & Kline, D. I. (2023). Effects of ocean acidification on dopamine‐mediated behavioral responses of a coral reef damselfish. Science of the Total Environment, 877, 162860. 10.1016/j.scitotenv.2023.162860 36931527

[jane70346-bib-0034] Hari, P. , Pumpanen, J. , Huotari, J. , Kolari, P. , Grace, J. , Vesala, T. , & Ojala, A. (2008). High‐frequency measurements of productivity of planktonic algae using rugged nondispersive infrared carbon dioxide probes. Limnology and Oceanography: Methods, 6, 347–354.

[jane70346-bib-0035] Hartig, F. (2018). DHARMa: residual diagnostics for hierarchical (multi‐level/mixed) regression models. R Packag version 020. https://cran.r‐project.org/package=DHARM

[jane70346-bib-0036] Holmes, T. H. , & McCormick, M. (2010). Soze‐selectivity of predatory reef fish on juvenile prey. Marine Ecology Progress Series, 399, 273–283.

[jane70346-bib-0037] Hönisch, B. , Ridgwell, A. , Schmidt, D. N. , Thomas, E. , Gibbs, S. J. , Sluijs, A. , Zeebe, R. , Kump, L. , Martindale, R. C. , Greene, S. E. , Kiessling, W. , Ries, J. , Zachos, J. C. , Royer, D. L. , Barker, S. , Marchitto, T. M. , Moyer, R. , Pelejero, C. , Ziveri, P. , … Williams, B. (2012). The geological record of ocean acidification. Science, 335, 1058–1063. 10.1126/science.1208277 22383840

[jane70346-bib-0038] Hulthén, K. , Chapman, B. B. , Nilsson, P. A. , Hansson, L.‐A. , Skov, C. , Brodersen, J. , Vinterstare, J. , & Brönmark, C. (2017). A predation cost to bold fish in the wild. Scientific Reports, 7, 1239. 10.1038/s41598-017-01270-w PMC5430796 28450699

[jane70346-bib-0039] Ishimatsu, A. , Hayashi, M. , Lee, K.‐S. , Kikkawa, T. , & Kita, J. (2005). Physiological effects on fishes in a high‐CO_2_ world. Journal of Geophysical Research, 110, C09S09. 10.1029/2004JC002564

[jane70346-bib-0040] Jutfelt, F. , & Hedgärde, M. (2013). Atlantic cod actively avoid CO_2_ and predator odour, even after long‐term CO_2_ exposure. Frontiers in Zoology, 10, 81. 10.1186/1742-9994-10-81 PMC3880174 24373523

[jane70346-bib-0041] Kay, M. (2024). tidybayes: Tidy data and GEOMS for bayesian models. R package version 3.0.7. http://mjskay.github.io/tidybayes/

[jane70346-bib-0042] Killen, S. S. , Adriaenssens, B. , Marras, S. , Claireaux, G. , & Cooke, S. J. (2016). Context dependency of trait repeatability and its relevance for management and conservation of fish populations. Conservation Physiology, 4, cow007. 10.1093/conphys/cow007 PMC4922260 27382470

[jane70346-bib-0043] Kroeker, K. J. , Kordas, R. L. , Crim, R. N. , & Singh, G. G. (2010). Meta‐analysis reveals negative yet variable effects of ocean acidification on marine organisms. Ecology Letters, 13, 1419–1434. 10.1111/j.1461-0248.2010.01518.x 20958904

[jane70346-bib-0044] Lenth, R. V. (2025). emmeans: Estimated marginal means, aka least‐squares means. R package version 1.10.7. https://rvlenth.github.io/emmeans/

[jane70346-bib-0045] Lüthi, D. , Le Floch, M. , Bereiter, B. , Blunier, T. , Barnola, J.‐M. , Siegenthaler, U. , Raynaud, D. , Jouzel, J. , Fischer, H. , Kawamura, K. , & Stocker, T. F. (2008). High‐resolution carbon dioxide concentration record 650,000–800,000 years before present. Nature, 453, 379–382. 10.1038/nature06949 18480821

[jane70346-bib-0046] Martin, J. G. A. , & Réale, D. (2008). Temperament, risk assessment and habituation to novelty in eastern chipmunks, *Tamias striatus* . Animal Behaviour, 75, 309–318.

[jane70346-bib-0047] McCormick, M. I. , Watson, S.‐A. , Simpson, S. D. , & Allan, B. J. M. (2018). Effect of elevated CO_2_ and small boat noise on the kinematics of predator‐prey interactions. Proceedings of the Royal Society B: Biological Sciences, 285, 20172650. 10.1098/rspb.2017.2650 PMC5897633 29563262

[jane70346-bib-0048] Midway, S. R. , Hasler, C. T. , Wagner, T. , & Suski, C. D. (2017). Predation of freshwater fish in environments with elevated carbon dioxide. Marine and Freshwater Research, 68, 1585–1592.

[jane70346-bib-0049] Mitchell, A. , Booth, D. J. , & Nagelkerken, I. (2022). Ocean warming and acidification degrade shoaling performance and lateralization of novel tropical–temperate fish shoals. Global Change Biology, 28, 1388–1401. 10.1111/gcb.16022 34918444

[jane70346-bib-0050] Mitchell, D. J. , Dujon, A. M. , Beckmann, C. , & Biro, P. A. (2019). Temporal autocorrelation: A neglected factor in the study of behavioral repeatability and plasticity. Behavioral Ecology, 31, 222–231.

[jane70346-bib-0051] Munday, P. L. , Cheal, A. J. , Dixson, D. L. , Rummer, J. L. , & Fabricius, K. E. (2014). Behavioural impairment in reef fishes caused by ocean acidification at CO_2_ seeps. Nature Climate Change, 4, 487–492.

[jane70346-bib-0052] Munday, P. L. , Dixson, D. L. , McCormick, M. I. , Meekan, M. , Ferrari, M. C. O. , & Chivers, D. P. (2010). Replenishment of fish populations is threatened by ocean acidification. Proceedings of the National Academy of Sciences, 107, 12930–12934. 10.1073/pnas.1004519107 PMC2919925 20615968

[jane70346-bib-0053] Munday, P. L. , McCormick, M. I. , Meekan, M. , Dixson, D. L. , Watson, S.‐A. , Chivers, D. P. , & Ferrari, M. C. (2012). Selective mortality associated with variation in CO_2_ tolerance in a marine fish. Ocean Acidification, 1, 1–5.

[jane70346-bib-0054] Munday, P. L. , Pratchett, M. S. , Dixson, D. L. , Donelson, J. M. , Endo, G. G. , Reynolds, A. D. , & Knuckey, R. (2013). Elevated CO_2_ affects the behavior of an ecologically and economically important coral reef fish. Marine Biology, 160, 2137–2144.

[jane70346-bib-0055] Nakagawa, S. , & Schielzeth, H. (2010). Repeatability for Gaussian and non‐Gaussian data: A practical guide for biologists. Biological Reviews, 85, 935–956. 10.1111/j.1469-185X.2010.00141.x 20569253

[jane70346-bib-0056] O'Dea, R. E. , Noble, D. W. A. , & Nakagawa, S. (2022). Unifying individual differences in personality, predictability and plasticity: A practical guide. Methods in Ecology and Evolution, 13, 278–293.

[jane70346-bib-0057] O'Dea, R. E. , Parker, T. H. , Chee, Y. E. , Culina, A. , Drobniak, S. M. , Duncan, D. H. , Fidler, F. , Gould, E. , Ihle, M. , Kelly, C. D. , Lagisz, M. , Roche, D. G. , Sánchez‐Tójar, A. , Wilkinson, D. P. , Wintle, B. C. , & Nakagawa, S. (2021). Towards open, reliable, and transparent ecology and evolutionary biology. BMC Biology, 19, 68. 10.1186/s12915-021-01006-3 PMC8034279 33836762

[jane70346-bib-0058] R Core Team . (2023). R: A language and environment for statistical computing. R Foundation for Statistical Computing.

[jane70346-bib-0059] Riebesell, U. , Fabry, V. J. , Hansson, L. , & Gattuso, J.‐P. (2011). Guide to best practices for ocean acidification research and data reporting. Publications Office of the European Union. 10.2777/66906

[jane70346-bib-0060] Roche, D. G. , Careau, V. , & Binning, S. A. (2016). Demystifying animal ‘personality’ (or not): Why individual variation matters to experimental biologists. Journal of Experimental Biology, 219, 3832–3843. 10.1242/jeb.146712 27852750

[jane70346-bib-0061] Roche, D. G. , Raby, G. D. , Norin, T. , Ern, R. , Scheuffele, H. , Skeeles, M. , Morgan, R. , Andreassen, A. H. , Clements, J. C. , Louissaint, S. , Jutfelt, F. , Clark, T. D. , & Binning, S. A. (2022). Paths towards greater consensus building in experimental biology. Journal of Experimental Biology, 225, jeb243559. 10.1242/jeb.243559 35258604

[jane70346-bib-0062] Roche, D. G. , Sundin, J. , Speers‐Roesch, B. , Nakagawa, S. , Clark, T. D. , & Binning, S. A. (2026). Data from: No effect of short‐term ocean acidification on individual‐level variation in behaviour and susceptibility to predation in a Great Barrier Reef damselfish. The Open Science Framework 10.17605/OSF.IO/CZVSF PMC13731831 42847619

[jane70346-bib-0063] Stoffel, M. A. , Nakagawa, S. , & Schielzeth, H. (2017). rptR: Repeatability estimation and variance decomposition by generalized linear mixed‐effects models. Methods in Ecology and Evolution, 8, 1639–1644.

[jane70346-bib-0064] Sundin, J. (2023). The effects of ocean acidification on fishes‐ history and future outlook. Journal of Fish Biology, 103, 765–772. 10.1111/jfb.15323 36648022

[jane70346-bib-0065] Taylor, E. B. , & McPhail, J. D. (1985). Burst swimming and size‐related predation of newly emerged coho salmon *Oncorhynchus kisutch* . Transactions of the American Fisheries Society, 114, 546–551.

[jane70346-bib-0066] Therneau, T. (2020). A Package for Survival Analysis in R. https://CRAN.R‐project.org/package=survival

[jane70346-bib-0067] Tresguerres, M. , & Hamilton, T. J. (2017). Acid–base physiology, neurobiology and behaviour in relation to CO_2_‐induced ocean acidification. Journal of Experimental Biology, 220, 2136–2148. 10.1242/jeb.144113 28615486

[jane70346-bib-0068] Tuomainen, U. , & Candolin, U. (2011). Behavioural responses to human‐induced environmental change. Biological Reviews, 86, 640–657. 10.1111/j.1469-185X.2010.00164.x 20977599

[jane70346-bib-0069] Vail, A. L. , & McCormick, M. I. (2011). Metamorphosing reef fishes avoid predator scent when choosing a home. Biology Letters, 7, 921–924. 10.1098/rsbl.2011.0380 PMC3210664 21653563

[jane70346-bib-0070] White, J. R. , Meekan, M. G. , McCormick, M. I. , & Ferrari, M. C. O. (2013). A comparison of measures of boldness and their relationships to survival in young fish. PLoS One, 8, e68900. 10.1371/journal.pone.0068900 PMC3712919 23874804

[jane70346-bib-0071] Wickham, H. (2016). ggplot2: Elegant graphics for data analysis. Springer‐Verlag.

[jane70346-bib-0072] Wolf, M. , & Weissing, F. J. (2012). Animal personalities: Consequences for ecology and evolution. Trends in Ecology & Evolution, 27, 452–461. 10.1016/j.tree.2012.05.001 22727728

