# Supplementary material for: No effect of short‐term exposure to ocean acidification on individual‐level variation in behaviour and susceptibility to predation in a Great Barrier Reef damselfish
Source: J Anim Ecol. 2026 Oct 8;95(11):2263–76. doi: 10.1111/1365-2656.70346 (PMC13731831; doi:10.1111/1365-2656.70346)

SUPPLEMENTARY MATERIAL

No effect of short-term exposure to ocean acidification on individual-level variation in behaviour and susceptibility to predation in a Great Barrier Reef damselfish

Table S1. Summary table with estimates, standard errors (SE), Z and *P* values for fixed effects included in a Poisson generalized linear mixed-effects model examining differences in number of predatory attacks that resulted in the capture of Ambon damsel (*Pomacentrus amboinensis*) in ambient CO_2 (N=28) and elevated CO_2 conditions (N=30). The sample size excludes Ambon damsel that were not captured. Predators (*Cephalopholis microprion*) were exposed to the same conditions of water chemistry as their prey. Predator identity was included as a random effect.

**Explanatory variable**	**Estimate**	**SE**	**Z**	***P***
Prey standard length	0.147	0.067	2.210	0.027
Mean emergence time	0.017	0.075	0.223	0.823
Mean time active	0.168	0.108	1.555	0.120
Mean time in/near shelter	0.490	0.243	2.012	0.044
Mean time displaying thigmotaxis	0.260	0.146	1.785	0.074
Mean time near novel object	0.386	0.137	2.815	0.005
Treatment_control	-0.040	0.182	-0.219	0.827

Figure S1. Graphical representation of the experimental timeline used to examine the effect of exposure to end-of-century CO_2 levels on personality and susceptibility to predation in *Pomacentrus amboinensis*. Fish personality was assessed by repeatedly measuring behaviour in the same fish in a novel environment assay on specific days (orange arrows) during 4 days exposure to present day (ambient) CO_2 (control, n=74) followed the next day by treatment with either present day CO_2 or elevated/high (end-of-century) CO_2 for 6 days (n=37 each). At the end of the CO_2 treatments, each fish was exposed to a predator-prey trial (using *Cephalopholis microprion* as predator) under the same CO_2 treatment.

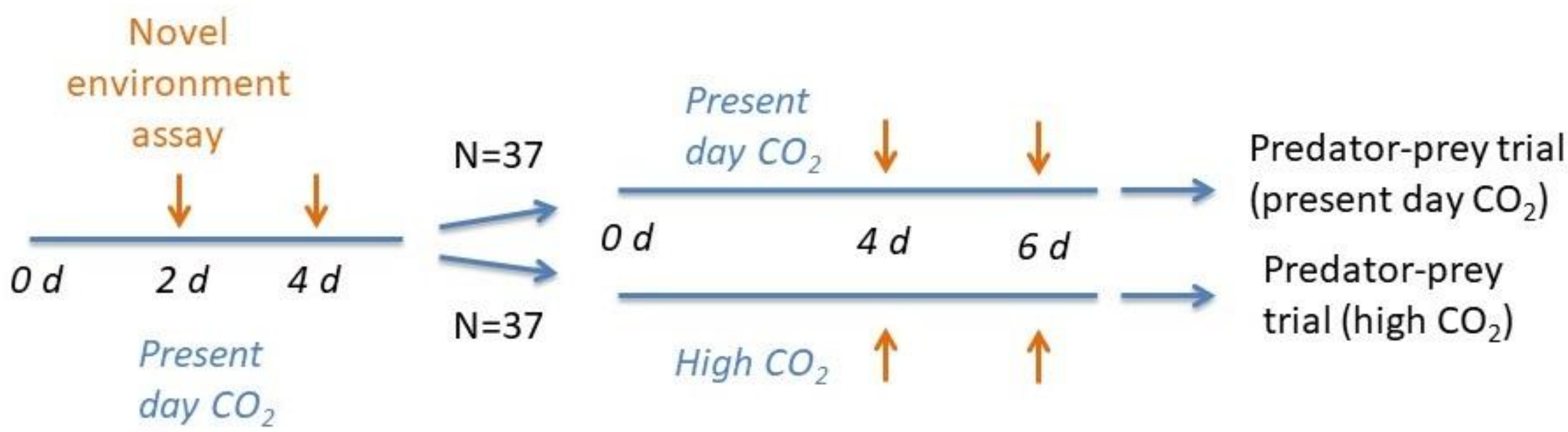

Figure S2. The distribution of model residuals (m_) after the following transformations: time to emergence from the shelter (emergT) was log-transformed; activity levels (activity) and thigmotaxis (thigmotaxis) were square-root-transformed; time spent sheltering (shelter) was not transformed; and time near a novel (novel) object was log-transformed after adding 0.5.

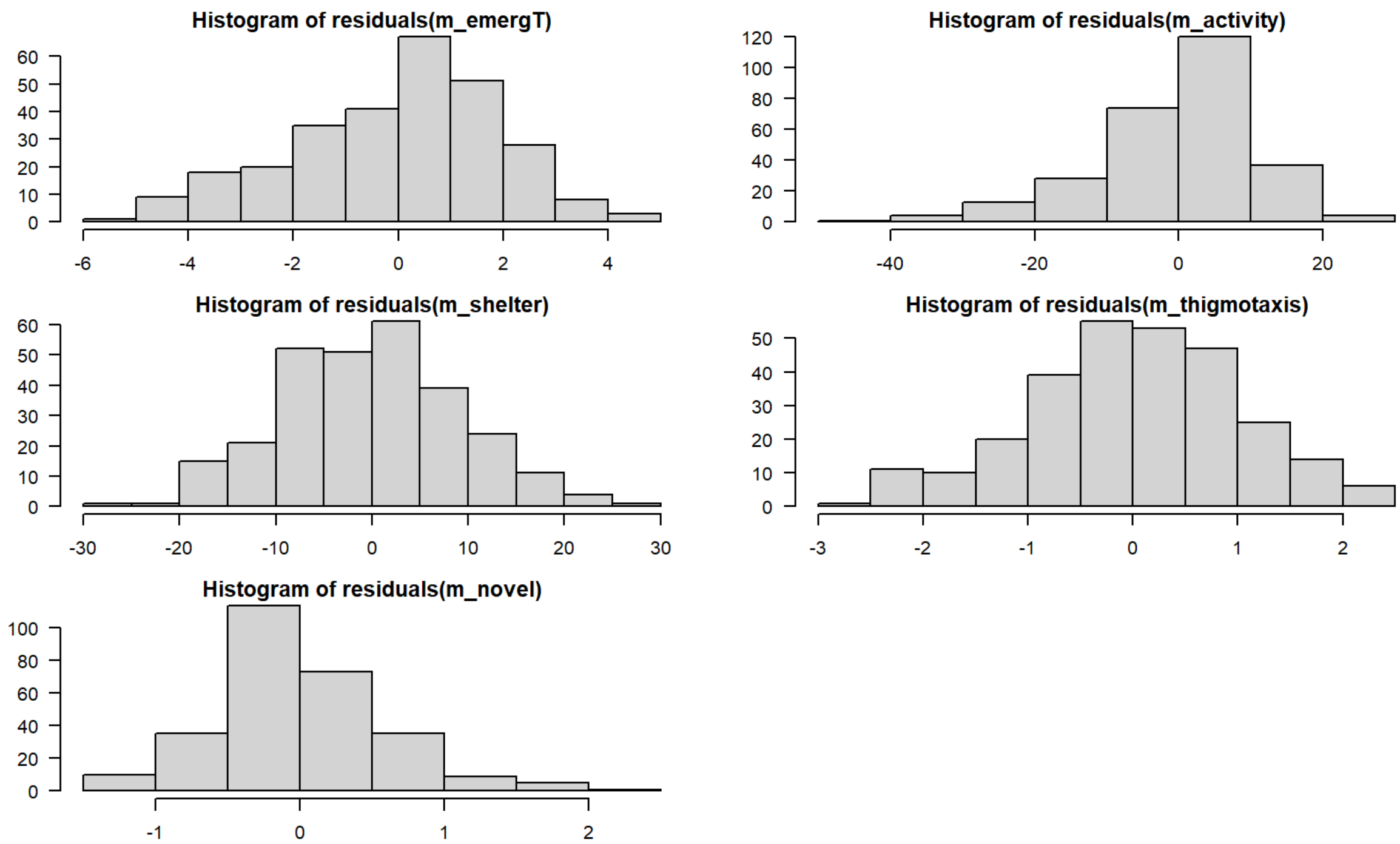

Figure S3. Reaction norm plots of non-transformed data for 5 behavioural measurements recorded across 4 trials for Ambon damselfish (*P. amboinensis*) exposed to present-day levels of dissolved CO_2 (ambient CO_2; n=36) and projected end-of-century CO_2-induced aquatic acidification (elevated CO_2, n=37): (A) emergence, (B) activity, (C) time sheltering, (D) thigmotaxis and (E) inspection of a novel object. Fish in the control group were exposed to present-day CO_2 levels in all 4 trials, whereas fish in the elevated CO_2 group were exposed to present-day CO_2 levels in trials 1-2 and projected end-of-century CO_2 levels in trials 3-4. Data points and coloured lines represent individual fish; the black line is the population-level (i.e., mean) response with the 95% CI credible interval indicated by dotted lines.

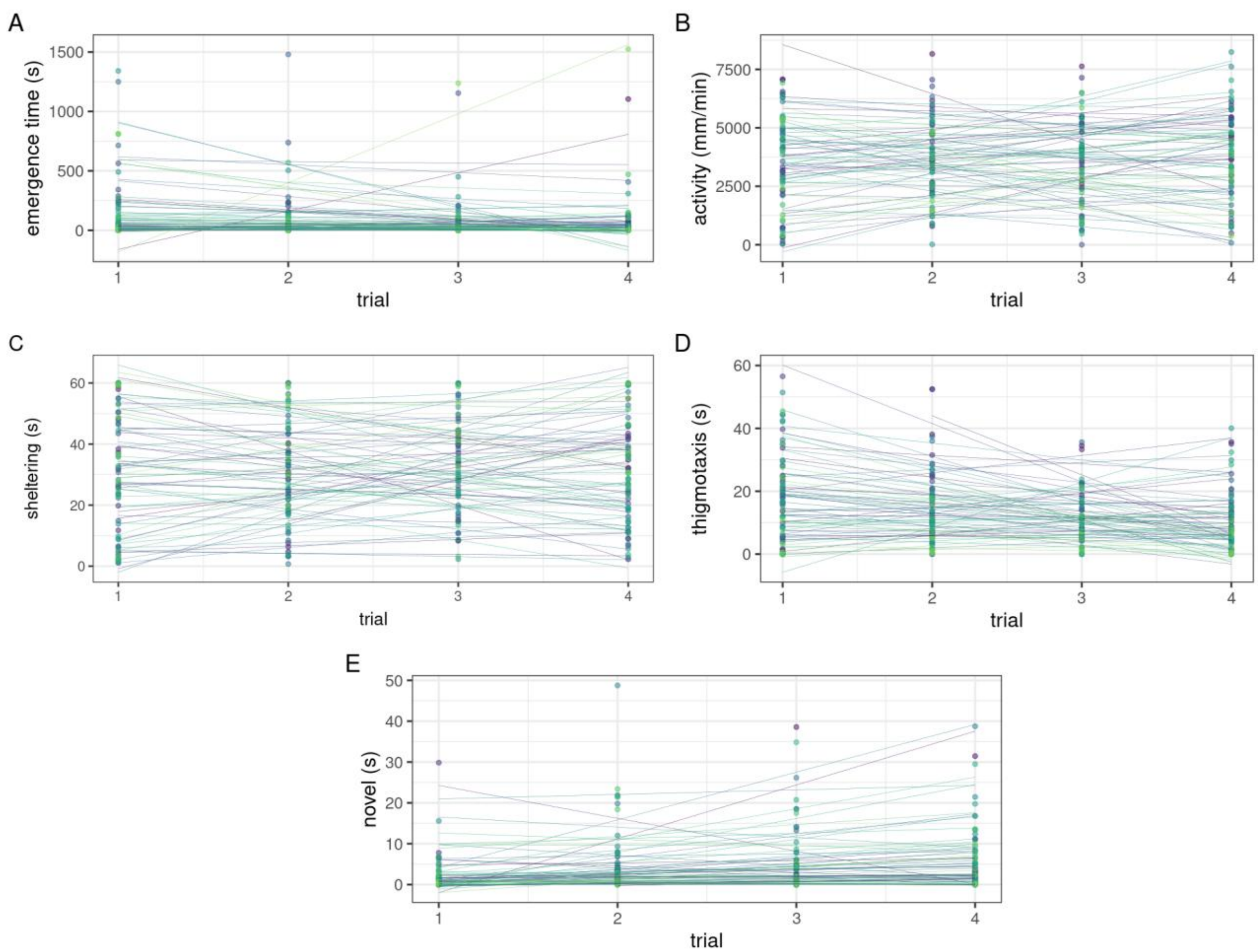

Figure S4. The number of predatory attacks by dot head rock cod (*Cephalopholis microprion*) resulting in the capture of Ambon damsel (*Pomacentrus amboinensis*) in ambient CO_2 (N=28) and elevated CO_2 (N=30) treatment groups. Ambon damsels that were not captured are not shown.

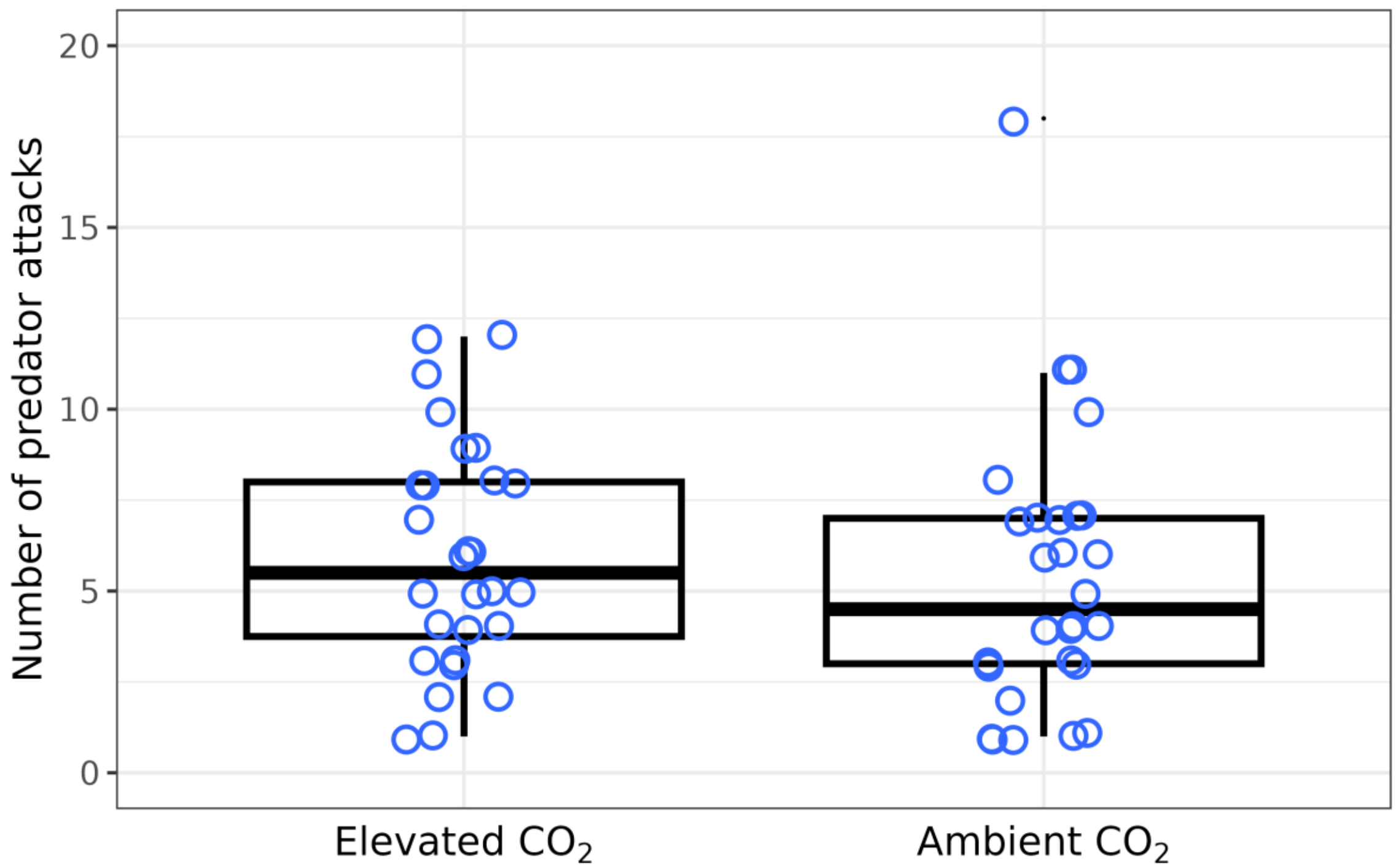

Supplement: Supplementary file 1 [file JANE-95-2263-s001.pdf]
